# Research on Kinematics and Stability of a Bionic Wall-Climbing Hexapod Robot

**DOI:** 10.1155/2019/6146214

**Published:** 2019-04-01

**Authors:** Shoulin Xu, Bin He, Heming Hu

**Affiliations:** Department of Control Science and Engineering, Tongji University, Shanghai 201804, China

## Abstract

Wall-climbing hexapod robot as a bionic robot has become a focus for extensive research, due to a wide range of practical applications. The most contribution of this paper is to analyze the kinematics and stability of a wall-climbing hexapod robot, so as to provide a theoretical basis for the stable walking and control of the robot on the wall. Firstly, the kinematics model of the wall-climbing hexapod robot is established based on the D-H method. Then, in order to keep the robot from tipping over, the stability of the wall-climbing hexapod robot is analyzed in depth, obtaining the critical condition which makes the robot to tip over. Afterward, the kinematics simulation of the wall-climbing hexapod robot is operated to analyze motion performances. Finally, the experiments are used to validate the proposed kinematics model and stability. The experimental results show that the kinematics model and stability condition of the wall-climbing hexapod robot are correct.

## 1. Introduction

With the development of robot technology, the application of robot has not been limited to the industrial field and gradually moved to more fields, such as service [1], medical treatment [2], and cleaning [3]. Wall-climbing robot as a bionic robot which movement flexibility, a variety of irregular terrain adaptability, and can cross obstacles, which can be widely used in the fields of building, traffic and disaster relief to complete testing, flaw detection, cleaning, rescue and other operations [4, 5].

At present, the main adhesive methods of wall-climbing robot include magnetic adhesion [6], adhesion of adhesive materials [7, 8], and vacuum adhesion [9]. The motion mechanism of robot mainly includes legged type [10, 11], crawler type [12], and frame type [13]. Legged-type robots offer strong obstacle crossing abilities and wall adaptability. The bearing capacity of crawler type is strong, but the turning is difficult. Frame structure is simple, but the motion is not continuous. In legged robots, the stability of hexapod robots is stronger than that of the biped robots and quadruped robots, and the control of hexapod robots is simple than that of the eight-legged robots.

Multilegged wall-climbing robot is a hybrid serial parallel mechanism. Many studies have studied the kinematics of a walking robot as a parallel mechanism. Howard et al. [14] proposed a kinematics model of a walking machine which was equivalent to a parallel mechanism and solved the inverse kinematics of the robot. Shkolnik and Tedrake [15] studied the Jacobian matrices of both the body and the swing legs of a quadruped robot. García-López et al. [16] presented a new kinematics model of a single leg of a hexapod robot, and the trajectory generation is implemented. To evaluate the leg movement performance, a simulator was developed in order to analyze the trajectory. Campa et al. [17] presented a procedure for computing the forward and inverse kinematics models of the hexapod robot. Xin et al. [18] proposed an extended hierarchical kinematic modeling method to derive the kinematic equations of the proposed hexapod robot. According to the kinematics model, the geometrical parameters of the leg are optimized utilizing a comprehensive objective function that considers both dexterity and payload. According to Soyguder and Alli [19], given the kinematics method of a hexapod robot was realized for walking, running, and bounding gaits, the developed kinematic makes both the system control easy and the system performance is improved by decreasing the run time.

Multilegged wall-climbing robot has a strong adaptability to the complex environment, but because of its foot end independent of each other, only to choose the appropriate landing point to ensure that the robot does not tipping over. Therefore, it is very important to study the stability of the robot, which is also an important reference for the design of the robot. With the deep research on the stability technology of the foot robot, the stability theory is maturing and the stability of the robot can be judged by various stability methods. Liu and Jiang [20] focused on the discussion of the stability of the bionic hexapod robot in the horizontal plane and on the slope by using the center of gravity of the projection method. Long et al. [21] proposed an improved force-angle stability margin measure method for a radial symmetrical hexapod robot under dynamic conditions. Roy and Pratihar [22] resented stability analysis based on normalized energy stability margin that is performed for turning motion of the robot with four duty factors for different angular strokes. Gui et al. [23] proposed a criterion called force-angle stability which is used to measure the performance of the robot which runs in complex environment with different gaits. Zhang et al. [24] presented the static stability of two kinds of tripod gait; when the step length of the robot meets certain conditions, the state of robot motion is statically stable. Sandoval-Castro et al. [25] proposed the normalized energy stability margin (SNE) criterion to analyze the robot stability.

In this paper, the most contribution is to establish the kinematics model and stability condition of a wall-climbing hexapod robot to provide a theoretical basis for the stable walking and control of the wall-climbing hexapod robot. The remainder of the paper is organized as follows. In Section 2, establishing the kinematics model of the wall-climbing hexapod robot based on the geometric model and the D-H method of the robot. In Section 3, the static stability condition of the wall-climbing hexapod robot is established, so as to obtain the critical condition of the robot tipping over. In Section 4, the kinematics simulation of the wall-climbing hexapod robot is carried out. In Section 5, the proposed kinematics model and stability condition are correct which are validated by the experiments.

## 2. Kinematics Model

### 2.1. Kinematics Model of One Leg

**Figure 1:**
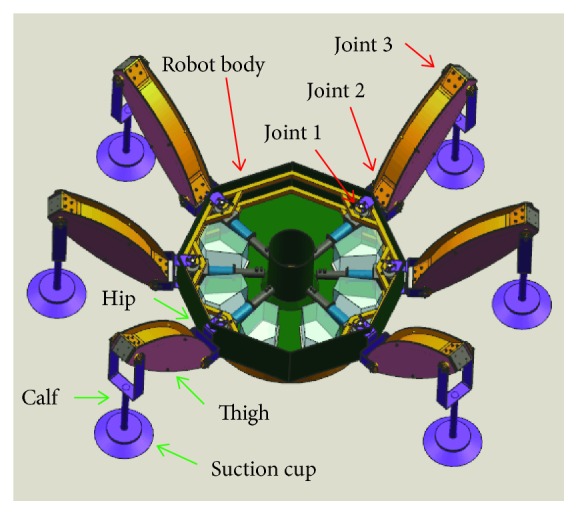
The CAD model of the wall-climbing hexapod robot.

First, a wall-climbing hexapod robot is designed, and the CAD model [26] is shown as Figure 1. The shape of the body frame of the wall-climbing hexapod robot is regular octagon, which is installed with the communication, control, energy, and other circuit systems. The wall-climbing hexapod robot is designed with six legs, and each leg consists of four components: suction cup, calf, thigh, and hip. The suction cup and the calf are connected by a spherical joint, while the calf and the thigh and the thigh and the hip are connected by revolute joints, which are parallel to the body. The hip and body are also connected via a revolute joint but is perpendicular to the body. The connection between the calf and the thigh is defined as joint 3, the connection between the thigh and the hip is defined as joint 2, and the connection between the hip and the body is defined as joint 1.

**Figure 2:**
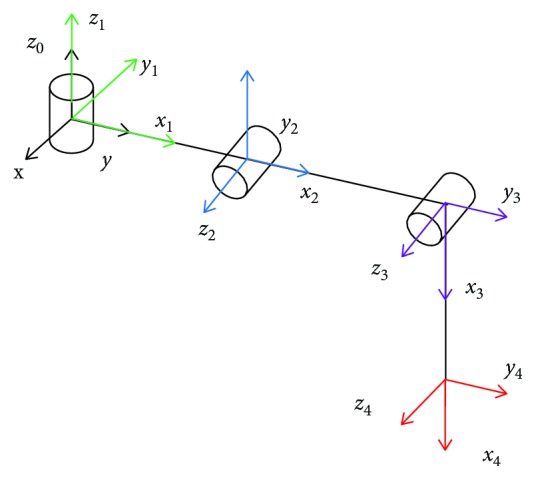
The D-H coordinate system of one leg.

When the wall-climbing hexapod robot adheres on the wall, it can be seen as a parallel mechanism and each leg can be equivalent to a three-link serial mechanism. Next, we first establish a forward kinematics model of one leg, and the geometry of one leg is shown in Figure 2.

Here, the D-H method is used to establish the forward kinematics of the wall-climbing hexapod robot. Supposing that *l*_*i*_ is the length of the joint *i*, *d*_*i*_ is the offset between the joint *i* − 1 and the joint *i* (moving joint), *α*_*i*_ is the twist angle of the joint *i*, and *θ*_*i*_ is the angle between the joints (revolving joints). The specific D-H parameters for one leg are shown in Table 1.

From the D-H method and Table 1 yields

(1) T40=c1c23−c1s23s1l3c1c23+c1l2c2+l1s1c23−s1s23−c1l3s1c23+s1l2c2+l1s23c230l3s23+l2s20001,

where

(2) ci=cosθi,si=sinθi,sij=sinθi+θj,cij=cosθi+θj.

So the coordinates of the end of the one leg are given as

(3) $$\begin{array}{c} \left[\begin{array}{c} p_{x_{0}}\\ p_{y_{0}}\\ p_{z_{0}} \end{array}\right]=\left[\begin{array}{c} l_{3}c_{1}c_{23}+c_{1}\left(l_{2}c_{2}+l_{1}\right)\\ l_{3}s_{1}c_{23}+s_{1}\left(l_{2}c_{2}+l_{1}\right)\\ l_{3}s_{23}+l_{2}s_{2} \end{array}\right]. \end{array}$$

In the following, on the basis of the kinematics model of one leg, the inverse kinematics of the one leg is analyzed.

Supposing

(4) T40=nxoxaxpx0nyoyaypy0nzozazpz00001,

(5) T40=T10∗T21∗T32∗T43.

Left multiply _1_^0^*T*^−1^ on both sides of equation (4) yields

(6) T−110∗T40=T−110∗T10∗T21∗T32∗T43=T21∗T32∗T43,

that is

(7) $$\begin{array}{c} \left[\begin{array}{cccc} c_{1}n_{x}+s_{1}n_{y} & c_{1}o_{x}+s_{1}o_{y} & c_{1}a_{x}+s_{1}a_{y} & c_{1}p_{x}+s_{1}p_{y_{0}}\\ -s_{1}n_{x}+c_{1}n_{y} & -s_{1}o_{x}+c_{1}o_{y} & -s_{1}a_{x}+c_{1}a_{y} & -s_{1}p_{x}+c_{1}p_{y_{0}}\\ n_{z} & o_{z} & a_{z} & p_{z_{0}}\\ 0 & 0 & 0 & 1 \end{array}\right]=\left[\begin{array}{cccc} c_{23} & -s_{23} & 0 & l_{3}c_{23}+l_{2}c_{2}+l_{1}\\ 0 & 0 & -1 & 0\\ s_{23} & c_{23} & 0 & l_{2}s_{23}+l_{2}s_{2}\\ 0 & 0 & 0 & 1 \end{array}\right]. \end{array}$$

Taking the second row and fourth column of the two matrices in equation (7), obtains

(8) $$\begin{array}{c} -s_{1}p_{x_{0}}+c_{1}p_{y_{0}}=0. \end{array}$$

By equation (8), yields

(9) $$\begin{array}{c} \theta_{1}=\arccos\left(\frac{p_{x_{0}}}{\sqrt{{p_{x_{0}}}^{2}+{p_{y_{0}}}^{2}}}\right). \end{array}$$

If the two sides of equation (5) are left multiply _2_^1^*T*^−1^_1_^0^*T*^−1^, obtains

(10) T−121T−110∗T40=T−121T−110∗T10∗T21∗T32∗T43=T32∗T43,

that is

(11) $$\begin{array}{c} \left[\begin{array}{cccc} f_{1}\left(n\right) & f_{1}\left(o\right) & f_{1}\left(a\right) & c_{2}\left(c_{1}p_{x_{0}}+s_{1}p_{y_{0}}\right)+s_{2}p_{z_{0}}-l_{1}c_{2}\\ f_{2}\left(n\right) & f_{2}\left(o\right) & f_{2}\left(a\right) & -s_{2}\left(c_{1}p_{x_{0}}+s_{1}p_{y_{0}}\right)+c_{2}p_{z_{0}}+l_{1}s_{2}\\ f_{3}\left(n\right) & f_{3}\left(o\right) & f_{3}\left(a\right) & s_{1}p_{x_{0}}-c_{1}p_{y_{0}}\\ 0 & 0 & 0 & 1 \end{array}\right]=\left[\begin{array}{cccc} c_{3} & -s_{3} & 0 & l_{3}c_{3}+l_{2}\\ s_{3} & c_{3} & 0 & l_{3}s_{3}\\ 0 & 0 & 1 & 0\\ 0 & 0 & 0 & 1 \end{array}\right], \end{array}$$

where

(12) $$\begin{array}{c} \left\{\begin{array}{c} f_{1}\left(n\right)=c_{2}\left(c_{1}n_{x}+s_{1}n_{y}\right)+s_{2}n_{z},\\ f_{2}\left(n\right)=-s_{2}\left(c_{1}n_{x}+s_{1}n_{y}\right)+c_{2}n_{z},\\ f_{3}\left(n\right)=s_{1}n_{x}-c_{1}n_{y}. \end{array}\right. \end{array}$$

Taking the first row and fourth column, the second row and fourth column of the two matrices in equation (11) obtains

(13) $$\begin{array}{c} \left\{\begin{array}{c} c_{2}\left(c_{1}p_{x_{0}}+s_{1}p_{y_{0}}\right)+s_{2}p_{z_{0}}-l_{1}c_{2}=l_{3}c_{3}+l_{2},\\ -s_{2}\left(c_{1}p_{x_{0}}+s_{1}{\text{p}}_{y_{0}}\right)+c_{2}p_{z_{0}}+l_{1}s_{2}=l_{3}s_{3}, \end{array}\right. \end{array}$$

Solving equation (13) yields

(14) $$\begin{array}{c} \theta_{2}=-\arctan\frac{L*p_{z_{0}}+l_{3}*\sin\left(\theta_{3}\right)*N}{l_{3}*p_{z_{0}}*\sin\left(\theta_{3}\right)-L*N},\\ \theta_{3}=\arccos∙\frac{l_{2}^{2}+l_{3}^{2}-p_{z_{0}}^{2}-M}{2*l_{2}*l_{3}}-\pi . \end{array}$$

Thus, obtains

(15) $$\begin{array}{c} \left\{\begin{array}{c} \theta_{1}=\arccos\left(\frac{p_{x0}}{\sqrt{{p_{x0}}^{2}+{p_{y0}}^{2}}}\right),\\ \theta_{2}=-\arctan\frac{L*p_{z0}+l_{3}*\sin\left(\theta_{3}\right)*N}{l_{3}*p_{z0}*\sin\left(\theta_{3}\right)-L*N},\\ \theta_{3}=\arccos\frac{l_{2}^{2}+l_{3}^{2}-p_{z0}^{2}-M}{2*l_{2}*l_{3}}-\pi , \end{array}\right. \end{array}$$

where

(16) $$\begin{array}{c} \left\{\begin{array}{c} L=l_{2}+l_{3}*\cos\left(-\theta_{3}\right),\\ M={\left(p_{x0}-l_{1}*\cos\left(\theta_{1}\right)\right)}^{2}+{\left(p_{y0}-l_{1}*\sin\left(\theta_{1}\right)\right)}^{2},\\ N=\sqrt{M}. \end{array}\right. \end{array}$$

### 2.2. Kinematics Model of the Body

**Figure 3:**
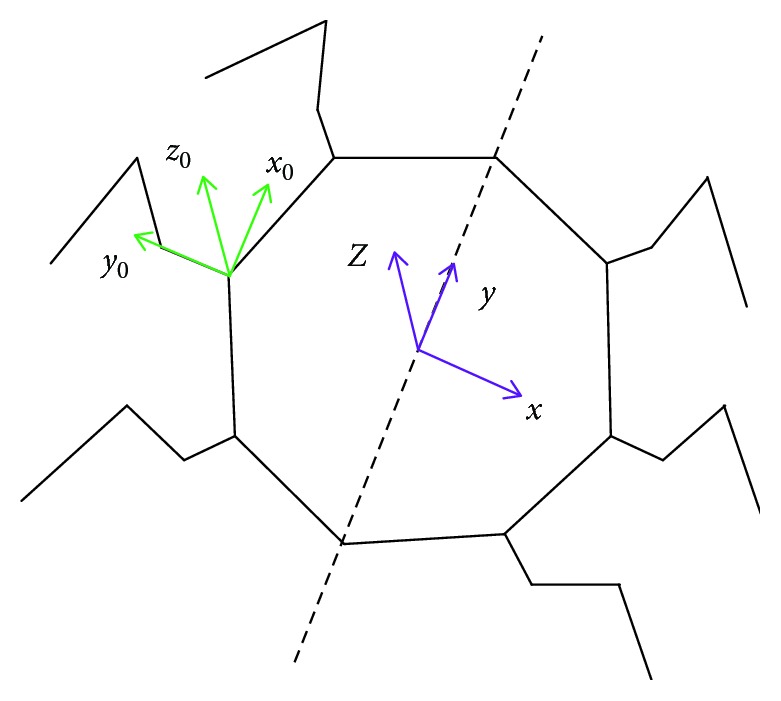
The body coordinate system and the leg coordinate system.

On the basis of establishing the forward kinematics of one leg, next, by coordinate transformation, the position relation between the center of the body and the end of the leg is obtained. The position relation between the body coordinate system and the leg coordinate system is shown in Figure 3.

The coordinates of the end of the leg in the body coordinate system can be obtained by coordinate transformation; the coordinate transformation is given as

(17) $$\begin{array}{c} X=r+R*X_{0}, \end{array}$$

where *X* is the body coordinate system, *r* is the transformation matrix of the position coordinate system, *R* is the transformation matrix of the direction coordinate system, and *X*_0_ is the single leg base system.

From Figure 3, yields

(18) $$\begin{array}{c} r=\left[\begin{array}{c} -350\\ 0\\ 0 \end{array}\right], \end{array}$$

(19) $$\begin{array}{c} R=\left[\begin{array}{ccc} \cos\theta & -\sin\theta & 0\\ \sin\theta & \cos\theta & 0\\ 0 & 0 & 1 \end{array}\right]=\left[\begin{array}{ccc} 0 & -1 & 0\\ 1 & 0 & 0\\ 0 & 0 & 1 \end{array}\right] \end{array}$$

Substituting equations (18) and (19) into equation (17) obtains

(20) $$\begin{array}{c} X=r+R*X_{0}=\left[\begin{array}{c} -350\\ 0\\ 0 \end{array}\right]+\left[\begin{array}{ccc} 0 & -1 & 0\\ 1 & 0 & 0\\ 0 & 0 & 1 \end{array}\right]*\left[\begin{array}{c} l_{3}c_{1}c_{23}+c_{1}\left(l_{2}c_{2}+l_{1}\right)\\ l_{3}s_{1}c_{23}+s_{1}\left(l_{2}c_{2}+l_{1}\right)\\ l_{3}s_{23}+l_{2}s_{2} \end{array}\right]=\left[\begin{array}{c} -l_{3}s_{1}c_{23}-s_{1}\left(l_{2}c_{2}+l_{1}\right)-350\\ l_{3}c_{1}c_{23}+c_{1}\left(l_{2}c_{2}+l_{1}\right)\\ l_{3}s_{1}c_{23}+l_{2}s_{2} \end{array}\right]. \end{array}$$

Thus, yields

(21) $$\begin{array}{c} \left[\begin{array}{c} p_{x}\\ p_{y}\\ p_{z} \end{array}\right]=\left[\begin{array}{c} -l_{3}s_{1}c_{23}-s_{1}\left(l_{2}c_{2}+l_{1}\right)-350\\ l_{3}c_{1}c_{23}+c_{1}\left(l_{2}c_{2}+l_{1}\right)\\ l_{3}s_{23}+l_{2}s_{2} \end{array}\right]. \end{array}$$

## 3. Static Stability Analysis

When wall-climbing hexapod robot walks on a vertical wall, it will be removed from the wall due to gravity. Through six vacuum suction cups which are fixed at the foot, the suction cup adhered on the wall, depending on the pressure difference between the inside and outside of the sucker. At this time, the traditional ZMP stability criterion [26–30] has no projection of the center of gravity on the contact surface; it is impossible to determine whether the robot is in stable state by means of projection and support. The wall-climbing hexapod robot has three kinds of instability on the vertical wall, the vertical tipping instability, lateral tipping instability, and fall instability, respectively. The analysis of the three unstable states will help us to choose the appropriate adhesion force, while maintaining the robot safe and stable operation under the premise of as much as possible to reduce the robot's energy consumption. This paper assumes that the robot is always walking at low speed and at constant speed, regardless of acceleration. The triangular gait is the most common gait of the robot. Under the triangular gait, the robot has the highest walking speed and efficiency. Thus, the stability of the robot is analyzed under the triangular gait.

### 3.1. Analysis of the Adhesive Force at the Foot of the Robot

**Figure 4:**
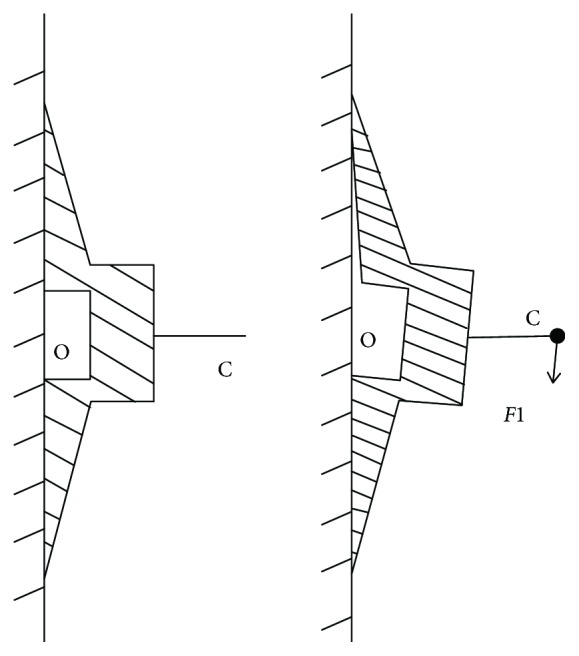
Force deformation diagram of the suction cup.

**Figure 5:**
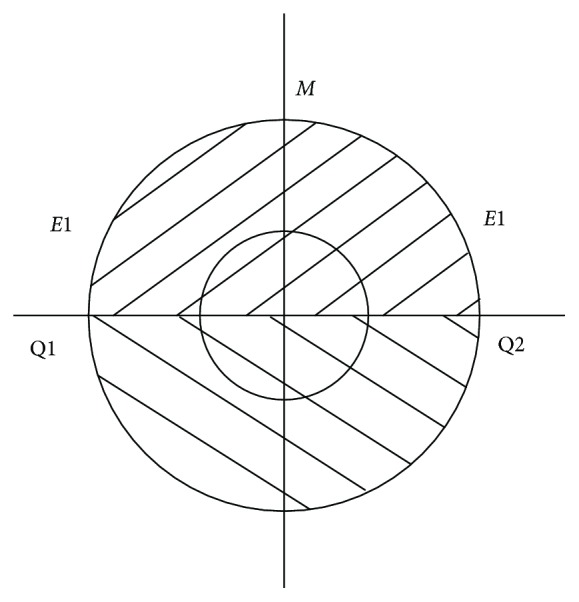
The tension and compression model of the suction cup.

A robot walks on the wall; each end of the suction cup is subjected to a tilting load which is parallel to the wall (the force acting on the central axis of the suction cup). Only the suction cup is not turned over; the robot will be safe to walk on the wall. When the end of the suction cup is subjected to the force acting on the central axis of the suction cup, the load is reversed vertically. The upper half of the suction cup is pulled and the lower half is squeezed, as shown in Figure 4. When the critical state of the suction cup is turned over, the suction cup appears on the *E*_1_*E*_2_ line where the force is greatest. As the bulge continues to grow, the suction cup will leak first from the point *M*, causing the entire suction cup to tip over, as shown in Figure 5.

Under the tilting load *F*_1_, the rod will deflect *γ* degrees downward. At this time, it will cause the suction cup corresponding uplift and then change the effective adhesion area *S* of the suction cup. Supposing that *D*_1_ is inside diameter of the suction cup and *D*_1_ = 150 mm and *D*_2_ is outside diameter of the suction cup and *D*_2_ = 250 mm, the relation between the effective adhesion area *S* and the deflection angle *γ* is obtained as follows:

(22) $$\begin{array}{c} S=\pi {\left(\frac{D_{2}}{2}\right)}^{2}-\left\{\sqrt{{\left(\frac{D_{2}}{2}\right)}^{2}-{\left(\frac{D_{1}}{2}\right)}^{2}}+\sqrt{{\left(\frac{D_{2}}{2}\right)}^{2}-{\left[\frac{D_{1}}{2}+D_{1}\sin\left(\frac{\gamma }{2}\right)\right]}^{2}}\right\}D_{1}\sin\left(\frac{\gamma }{2}\right)=49062.50-150*\left\{100+\sqrt{15625-{\left[75+150\sin\left(\frac{\gamma }{2}\right)\right]}^{2}}\right\}\sin\left(\frac{\gamma }{2}\right). \end{array}$$

The suction cup test shows that when the maximum *F*_1_ is 1926 N, the rod reaches the maximum deflection angle, at this time, *γ* = 28.2 degrees. In a very small range, the deflection angle *γ* and the end force *F*_1_ are approximated as a linear function; its relation is

(23) $$\begin{array}{c} F_{1}=68.30\gamma . \end{array}$$

**Figure 6:**
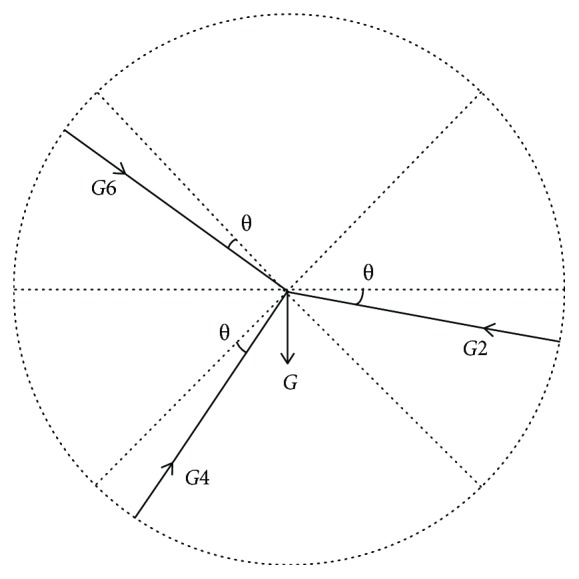
Force of state overturning loading.

The analysis shows that the force *F*_1_ is due to the gravity acting on the body, as shown in Figure 6.

The forces at the three suction cups are *G*_2_, *G*_4_, and *G*_6_, which satisfy the following equation:

(24) $$\begin{array}{c} \left\{\begin{array}{c} G_{6}\cos\left(45-\theta \right)+G_{4}\cos\left(45+\theta \right)=G_{2}\cos\theta ,\\ G_{6}\sin\left(45-\theta \right)+G=G_{4}\sin\left(45+\theta \right)+G_{2}\sin\theta ,\\ G_{6}l+Gl\sin\left(45-\theta \right)=G_{2}l\sin\left(45+2\theta \right). \end{array}\right. \end{array}$$

Solving equation (24) obtains

(25) $$\begin{array}{c} \left\{\begin{array}{c} G_{2}=G\frac{\cos\left(\theta_{1}\right)-\sin\left(\theta_{2}\right)\sin\left(\theta_{2}\right)\cos\left(\theta_{1}\right)-\sin\left(\theta_{1}\right)\sin\left(\theta_{2}\right)\cos\left(\theta_{2}\right)}{A},\\ G_{4}=G\frac{\cos\theta -\sin\left(\theta_{2}\right)\sin\left(\theta_{2}\right)\cos\theta -\sin\left(\theta_{3}\right)\cos\left(\theta_{2}\right)+\sin\theta \sin\left(\theta_{2}\right)\cos\left(\theta_{2}\right)}{A},\\ G_{6}=G\frac{\sin\left(\theta_{3}\right)\cos\left(\theta_{1}\right)-\sin\left(\theta_{1}\right)\sin\left(\theta_{2}\right)\cos\theta -\sin\theta \sin\left(\theta_{2}\right)\cos\left(\theta_{1}\right)}{A}, \end{array}\right. \end{array}$$

where

(26) $$\begin{array}{c} \left\{\begin{array}{c} \theta_{1}=45+\theta ,\\ \theta_{2}=45-\theta ,\\ \theta_{3}=45+2\theta ,\\ A=\sin\left(\theta_{1}\right)\cos\theta -\sin\left(\theta_{3}\right)\sin\left(\theta_{1}\right)\cos\left(\theta_{2}\right)-\sin\left(\theta_{3}\right)\sin\left(\theta_{2}\right)\cos\left(\theta_{1}\right)+\sin\theta \cos\left(\theta_{1}\right). \end{array}\right. \end{array}$$

**Figure 7:**
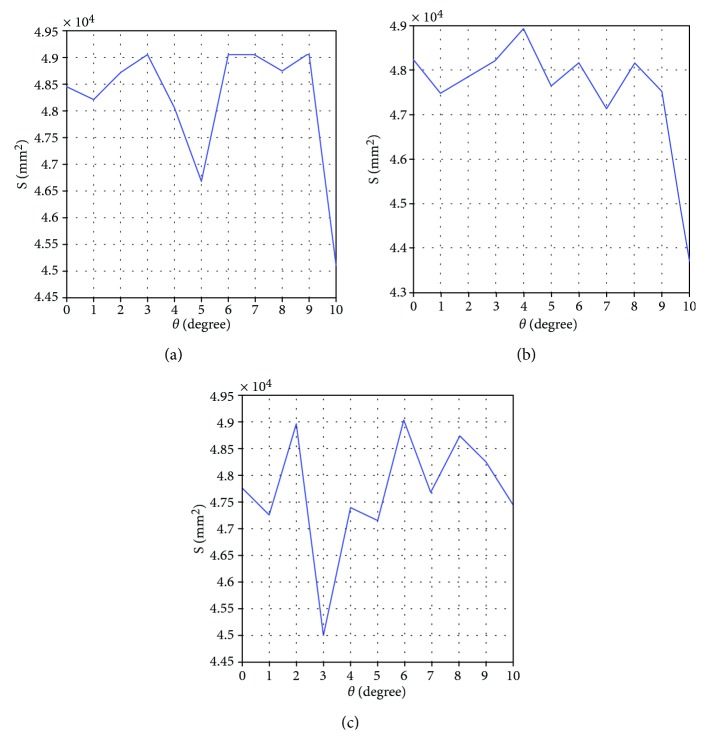
(a) Relation between the deflection angle *θ* and the effective adhesion area *S* of the leg 2. (b) Relation between the deflection angle *θ* and the effective adhesion area *S* of the leg 4. (c) Relation between the deflection angle *θ* and the effective adhesion area *S* of the leg 6.

To sum up, with the change of robot pose, the effective adhesion area of the suction cup will change. When the pressure difference between the inside and outside of the suction cup is a fixed value, the adhesive force on each suction cup can be changed. The relations between the deflection angle of the leg and the effective adhesion area *S* of the suction cup are shown in Figure 7.

### 3.2. Walking Stability Analysis of Robot

**Figure 8:**
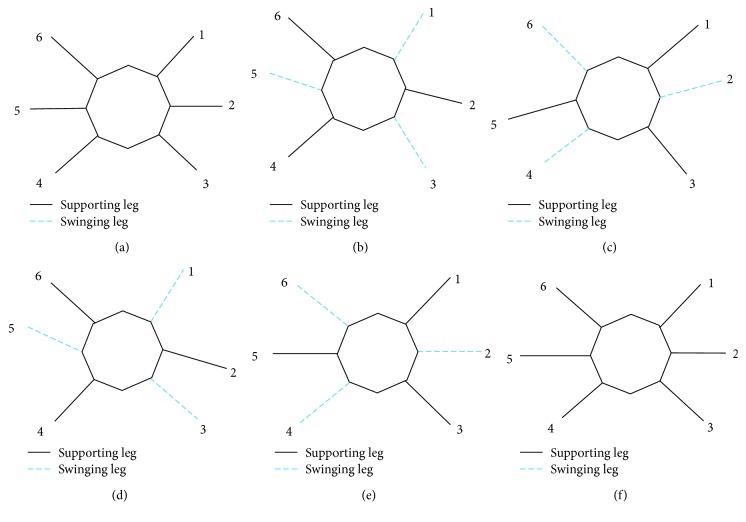
A general triangular gait of robot walks on the wall.

Here, supposing that the wall-climbing hexapod robot which selects a general triangular gait walks on the wall, as shown in Figure 8, it mainly includes the initial state represented by *a* and *f* and four intermediate states of *b*, *c*, *d*, and *e*.

**Figure 9:**
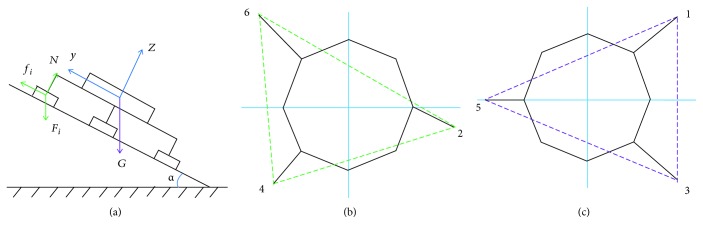
(a) Force analysis of the robot. (b) *b* status support of the robot. (c) *e* status support of the robot.

The following is a detailed analysis of the status *b* and *e*, as shown in Figure 9(a). The support polygons in the triangular gait are shown in Figures 9(b) and 9(c).

A longitudinal overturning instability analysis is performed on the *b* status support with the axis 24 as the tilting axis:

(27) $$\begin{array}{c} \left\{\begin{array}{c} F_{6}*l_{6}+G*\cos\alpha *l_{G}>G_{z24}*h,\\ \frac{F}{G}>0.113*\sin\alpha -0.352*\cos\alpha . \end{array}\right. \end{array}$$

Next, a longitudinal overturning instability analysis is performed on the *e* status support with the axis 35 as the tilting axis:

(28) $$\begin{array}{c} \left\{\begin{array}{c} F_{1}*l_{1}+G*\cos\alpha *l_{G}>G_{z35}*h,\\ \frac{F}{G}>0.116*\sin\alpha -0.294*\cos\alpha , \end{array}\right. \end{array}$$

where *G* is the total gravity of a robot and a load. *G*_*z*24_ is the gravity which causes the robot to rotate vertically around the axis 24. *G*_*z*35_ is the gravity which causes the robot to rotate vertically around the axis 35. *F*_*i*_ is the adhesive force and *F*_1_ = *F*_2_ = *F*_3_ = *F*_4_ = *F*_5_ = *F*_6_ = *F*. *l*_*G*_ is the distance from the center of gravity to the tilting axis of a robot. *l*_*i*_ is the distance from the center of the suction cup to the tilting axis. *h* is the distance between the centroid of robot and the wall. *α* is the inclination angle of the wall.

A lateral overturning instability analysis is performed on the *b* status support with the axis 46 as the tilting axis:

(29) $$\begin{array}{c} \left\{\begin{array}{c} F_{2}*l_{2}+G*\cos\alpha *l_{G}>G_{h46}*h,\\ \frac{F}{G}>0.010*\sin\alpha -0.420*\cos\alpha , \end{array}\right. \end{array}$$

where *G*_*h*46_ is the gravity which causes the robot to rotate vertically around the axis 46.

When the *e* status support takes the axis 13 as the tilting axis, the lateral overturning instability analysis shows that the component of gravity will not cause the robot to lateral overturning instability.

For *b* status support, to ensure that the robot does not slide on the wall, the balance conditions that need to be satisfied are as follows:

(30) $$\begin{array}{c} \left\{\begin{array}{c} \mu *\left(F_{2}+F_{4}+F_{6}+G*\cos\alpha \right)>G*\sin\alpha ,\\ \frac{F}{G}>0.417*\sin\alpha -0.333*\cos\alpha . \end{array}\right. \end{array}$$

For *e* status support, to ensure that the robot does not slide on the wall, the balance conditions that need to be satisfied are as follows:

(31) $$\begin{array}{c} \left\{\begin{array}{c} \mu *\left(F_{1}+F_{3}+F_{5}+G*\cos\alpha \right)>G*\sin\alpha ,\\ \frac{F}{G}>0.417*\sin\alpha -0.333*\cos\alpha , \end{array}\right. \end{array}$$

where *μ* is the friction coefficient between the wall and the suction cup, and *μ* = 0.8.

**Figure 10:**
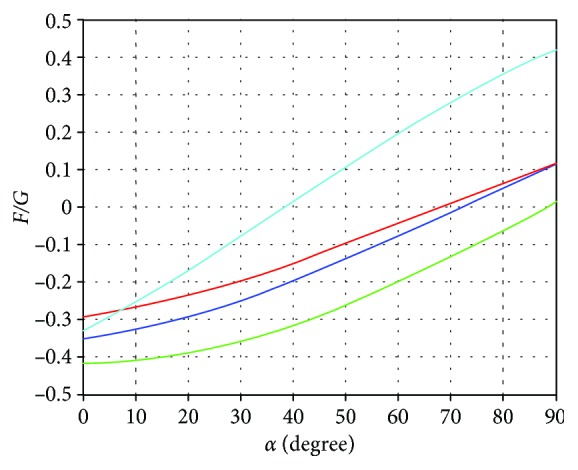
Instability critical curve of triangular gait.

Based on the above analysis, Figure 10 shows the instability critical curve of triangular gait. In Figure 10, the blue curve is the *b* status support longitudinal tipping instability curve, crossing the axis *x* at 72.29 degrees. The red curve is the *e* status support longitudinal tipping instability curve, crossing the axis *x* at 68.4 degrees. The green curve is the *b* status support lateral overturning instability curve, crossing the axis *x* at 88.67 degrees. The blue-green curve is the critical curve of the sliding instability of the robot, crossing the axis *x* at 38.52 degrees.

## 4. Model Simulations

### 4.1. Foot Trajectory of the Swinging Leg

In the triangular gait, the coordinates of the starting point, the highest point, and the falling point of the foot of the robot are (97.24, 551.49, -150) mm, (0, 599.95, -91.93) mm, and (-97.24, 551.49, -150) mm, respectively. The speed of the starting point is (0, 0, 0) mm/s, and the acceleration is (0, 0, 0) mm/s^2^. The rate of the falling point is (0, 0, 0) mm/s, and the acceleration is (0, 0, 0) mm/s^2^. The swing time is 4 s. Then, the foot trajectory of swinging leg is

(32) $$\begin{array}{c} \left\{\begin{array}{c} x\left(t\right)=-1.1395t^{5}+11.3953t^{4}-30.3875t^{3}+97.2400,\\ y\left(t\right)=-0.7572t_{6}+9.0863t^{5}-36.3450t^{4}+48.4600t^{3}+551.4900,\\ z\left(t\right)=-0.9073t^{6}+10.8881t^{5}-43.5525t^{4}+58.0700t^{3}-150.0000. \end{array}\right. \end{array}$$

**Figure 11:**
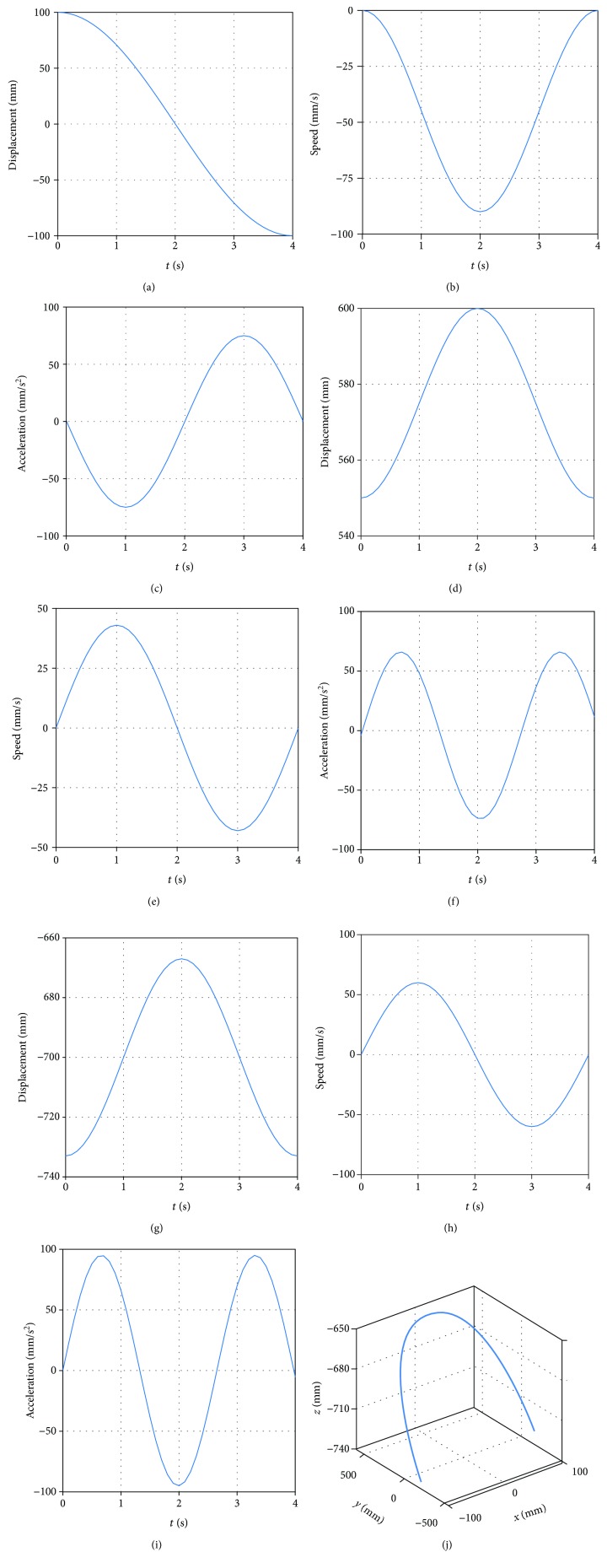
(a) The position of foot in the *x* direction. (b) The speed of foot in the *x* direction. (c) The acceleration of foot in the *x* direction. (d) The position of foot in the *y* direction. (e) The speed of foot in the *y* direction. (f) The acceleration of foot in the *y* direction. (g) The position of foot in the *z* direction. (h) The speed of foot in the *z* direction. (i) The acceleration of foot in the *z* direction. (j) Foot trajectory of swinging leg.

By equation (32), obtaining that the foot trajectory of swinging leg is shown in Figure 11.

### 4.2. Foot Trajectory of the Supporting Leg

On the triangular gait of the supporting leg, the intersection between the supporting leg and body as the origin, and establish the coordinate system. The coordinates of the starting point and the falling point of the foot of the robot are (-97.24, 551.49, -150) mm and (97.24, 551.49, -150) mm, respectively. The speed of the starting point is (0, 0, 0) mm/s, and the acceleration is (0, 0, 0) mm/s^2^. The rate of the falling point is (0, 0, 0) mm/s, and the acceleration is (0, 0, 0) mm/s^2^. The swing time is 4 s. Then, the foot trajectory of supporting leg is

(33) $$\begin{array}{c} \left\{\begin{array}{c} x\left(t\right)=1.1395t^{5}-11.3953t^{4}+30.3875t^{3}-97.2400,\\ y\left(t\right)=551.4900,\\ z\left(t\right)=-150.0000. \end{array}\right. \end{array}$$

**Figure 12:**
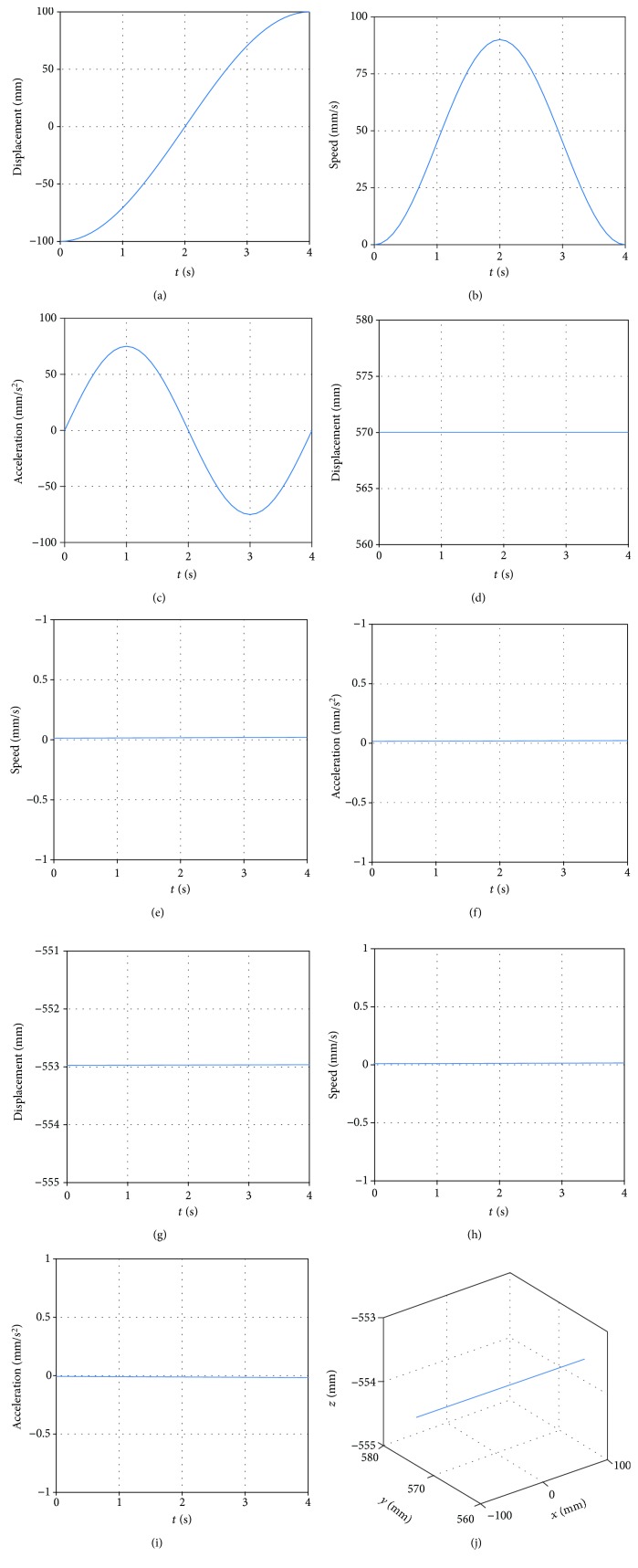
(a) The position of foot in the *x* direction. (b) The speed of foot in the *x* direction. (c) The acceleration of foot in the *x* direction. (d) The position of foot in the *y* direction. (e) The speed of foot in the *y* direction. (f) The acceleration of foot in the *y* direction. (g) The position of foot in the *z* direction. (h) The speed of foot in the *z* direction. (i) The acceleration of foot in the *z* direction. (j) Foot trajectory of the supporting leg.

By equation (33), obtaining that the foot trajectory of supporting leg is shown in Figure 12.

### 4.3. Motion Simulation of the Wall-Climbing Hexapod Robot

**Figure 13:**
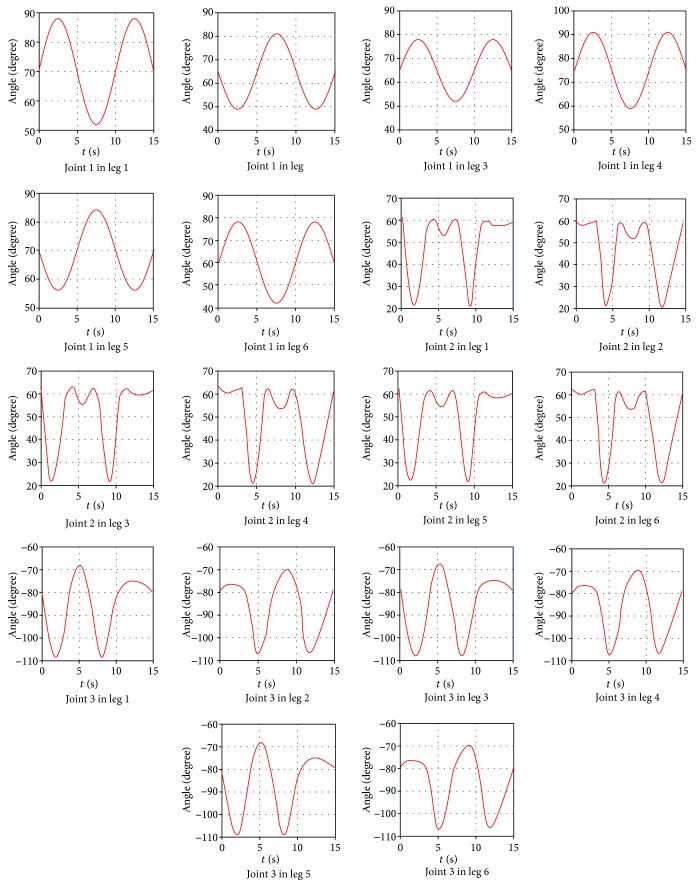
The driving function of the driving joint.

According to the foot trajectory planning and inverse kinematics, the relation between joint angle and time is obtained. Then, using the spline curve to drive the joint motion, the driving function is shown in Figure 13.

**Figure 15:**
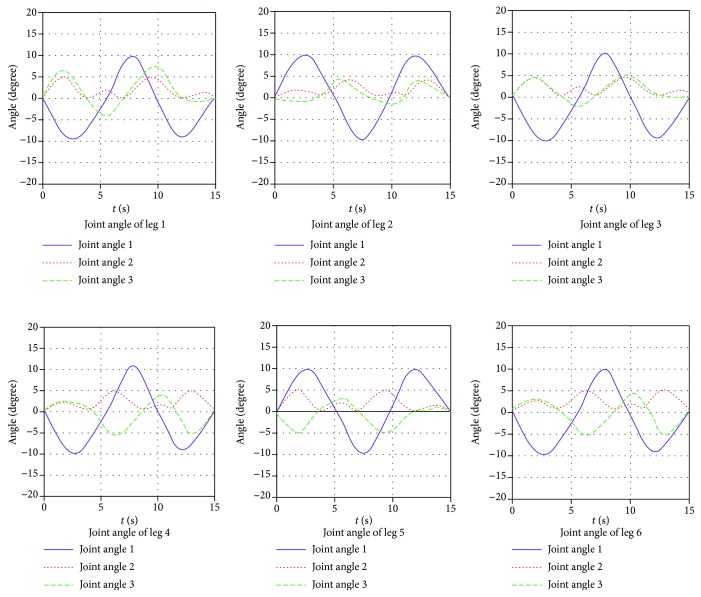
The joint angle change with time in triangular gait.

**Figure 16:**
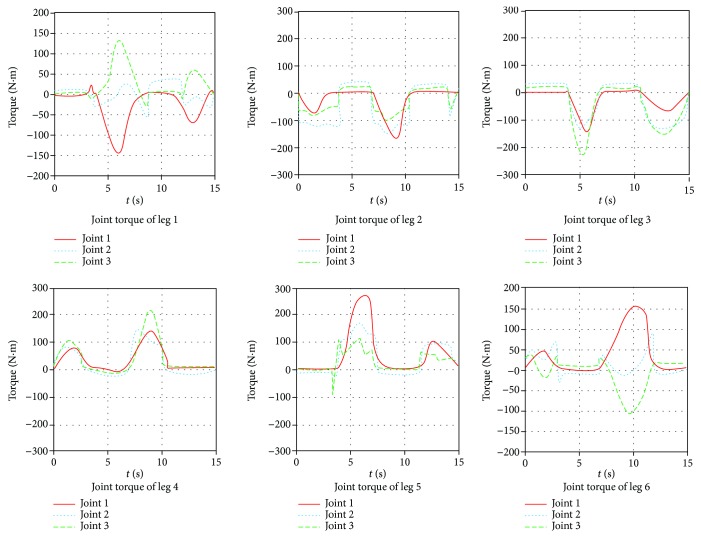
The joint torque change with time in triangular gait.

**Figure 17:**
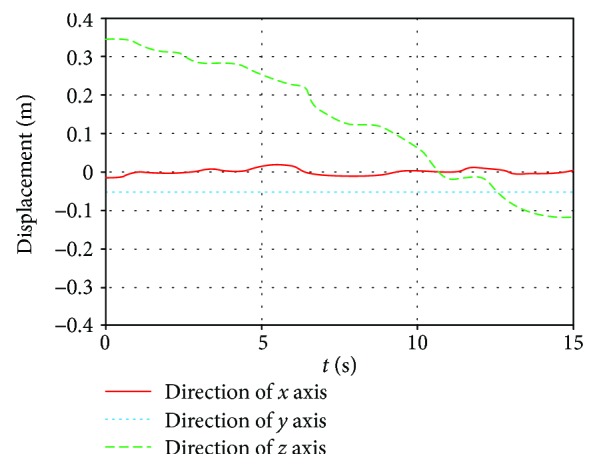
Motion simulation of the center of gravity displacement change with time in triangular gait.

Moreover, on the basis of the driving function, obtaining that the joint angle and torque change with time are shown in Figures 15 and 16. Meanwhile, motion simulation of the center of gravity displacement in the triangular gait is shown in Figure 17.

By Figure 15, it is obtained that the angular changes of each joint are continuous and gentle, without any angle mutation. In Figure 16, six legs are divided into 2 groups in the triangle gait, of which legs 1, 3, and 5 are one group and legs 2, 4, and 6 are another group. The torque variation of legs 1, 3, and 5 is the same, and the torque variation of legs 2, 4, and 6 is the same. The torque changes continuously as the robot runs, but torque changes occur at the time of each transition. By Figure 17, it is obtained that the robot moves along the *x* direction, and the displacement of the robot is 13.7 mm. The robot moves along the y direction, and the displacement of the robot is 1.3 mm. The robot runs with a body height of 150 mm, and offset is 0.87%; the robot can run smoothly. The robot moves along the *z* direction, and the displacement of the robot is 503.7 mm; the average speed is 30.9 mm/s.

## 5. Experiments

**Figure 18:**
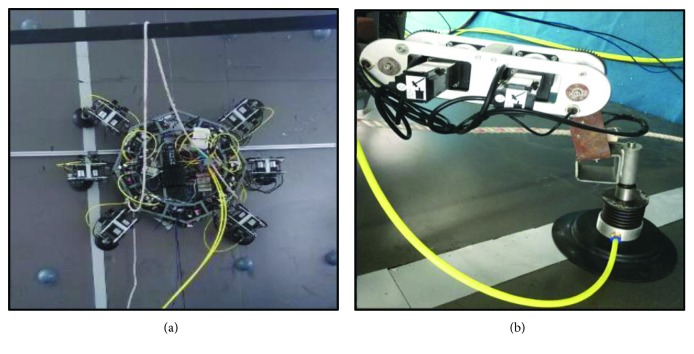
(a) The prototype of the wall-climbing hexapod robot. (b) The structure of one leg of the wall-climbing hexapod robot [31].

The kinematics and stability of the wall-climbing hexapod robot have been analyzed on the above. A prototype of the wall-climbing hexapod robot is developed, as shown in Figure 18. The motors and the retarders produced by Shenzhen Techservo Co. Ltd in China are selected. The suction cup which is produced by Japanese SMC Company is selected, of which the model is ZP2-250HTN. The motor model of joint 1 is ST8N40P10V2E, the motor model of joint 2 is ST8N40P20V2E, and the motor model of joint 3 is ST8N40P10V4E [26]. The retarder models of joints 1, 2, and 3 are S042L3-100 and RAD-60-2 [26]. The structural parameters of the robot are set as follows: *L*_1_ = 550 mm, *L*_2_ = 450 mm, *L*_3_ = 200 mm, and *r* = 350 mm. The total mass of the robot is 72.0 kg.

**Figure 19:**
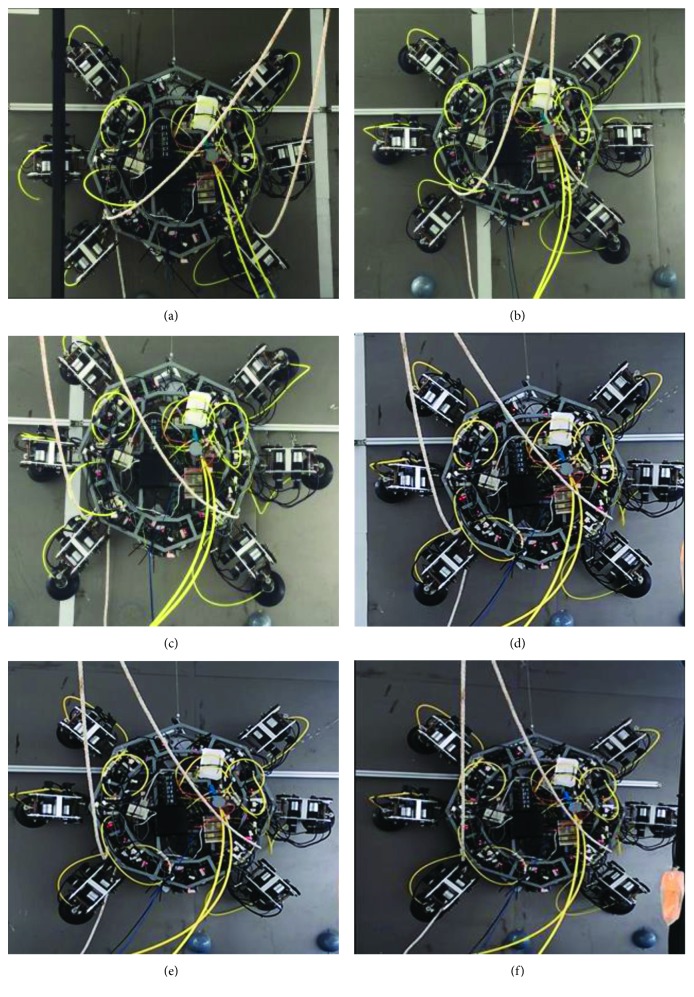
The wall-climbing hexapod robot walks on vertical wall with horizontal direction by using the triangular gait [31].

Next, the kinematics model and stability analysis of the wall-climbing hexapod robot are correct as shown in the experiments. The experimental results indicate that the kinematics models of one leg and body are correct; meanwhile, the experimental results are in good agreement with the kinematics simulation results. In addition, the experimental results show that the stability conditions of the wall-climbing hexapod robot are correct.  Figure 19 shows that the wall-climbing hexapod robot walks on the vertical wall with horizontal direction by using the triangular gait. The wall-climbing hexapod robot can walk well on the vertical wall, and the stability is well.

## 6. Conclusions and Future Work

In this paper, the most contribution is to analyze the kinematics and stability condition of the wall-climbing hexapod robot to provide a theoretical basis for the stable walking and control of the robot. First, the kinematics model of one leg is established, on the basis of the kinematics model, the inverse kinematics of the one leg is solved. Meanwhile, the kinematics model of the body is obtained by coordinate transformation. Second, to make the robot walk steadily on the wall, and no tipping occurs, the static stability of wall-climbing hexapod robot is analyzed, obtaining the critical stability condition of the robot. Third, the kinematics simulation of the wall-climbing hexapod robot is operated to analyze motion performances, obtaining the foot trajectory of the swinging and supporting legs. Moreover, the relation between joint angle and time and joint torque and time is obtained by simulation. Finally, the experiments are used to validate the proposed kinematics model and stability conditions. The experimental results show that the proposed kinematics model and stability conditions of the wall-climbing hexapod robot are correct.

In the future, we can establish a dynamic model of the wall-climbing hexapod robot, obtaining the relation between the driving force and acceleration, velocity, and position of the wall-climbing hexapod robot, so as to provide an effective basis for motion control and force control of the wall-climbing hexapod robot. In addition, the supporting legs which are deformed are influenced by the gravity of the body. The accuracy of motion control of the wall-climbing hexapod robot is thus affected, and the walking trajectory deviation occurs in the process. Thus, the stiffness of the wall-climbing hexapod robot is investigated in the future.

## Figures and Tables

**Figure 14:**
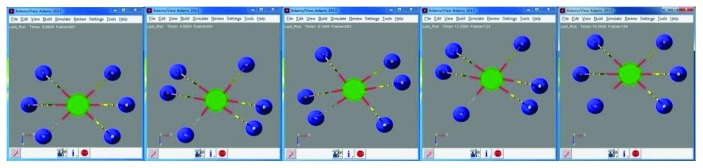
Motion simulation of the wall-climbing hexapod robot in triangular gait.

**Table 1:** The D-H parameters of one leg.

*l*_*i*_	*d*_*i*_	*α*_*i*_	*θ*_*i*_
0	0	0°	*θ*_1_
*l*_1_	0	90°	*θ*_2_
*l*_2_	0	0°	*θ*_3_
*l*_3_	0	0°	0

## Data Availability

The data used to support the findings of this study are available from the corresponding author upon request.

## References

[1] Han I. H., Yi H., Song C.-W., Jeong H. E., Lee S.-Y. (2017). A miniaturized wall-climbing segment robot inspired by caterpillar locomotion. *Bioinspiration & Biomimetics*.

[2] An C. Y., Syu J. H., Tseng C. S., Chang C. J. (2017). An ultrasound imaging-guided robotic HIFU ablation experimental system and accuracy evaluations. *Applied Bionics and Biomechanics*.

[3] Barnfather J. D., Goodfellow M. J., Abram T. (2017). Positional capability of a hexapod robot for machining applications. *The International Journal of Advanced Manufacturing Technology*.

[4] Zhong G., Chen L., Jiao Z., Li J., Deng H. (2018). Locomotion control and gait planning of a novel hexapod robot using biomimetic neurons. *IEEE Transactions on Control Systems Technology*.

[5] Rastegarpanah A., Saadat M., Borboni A. (2016). Parallel robot for lower limb rehabilitation exercises. *Applied Bionics and Biomechanics*.

[6] Tavakoli M., Lourenço J., Viegas C., Neto P., de Almeida A. T. (2016). The hybrid OmniClimber robot: wheel based climbing, arm based plane transition, and switchable magnet adhesion. *Mechatronics*.

[7] Sato E., Iki S., Yamanishi K., Horibe H., Matsumoto A. (2017). Dismantlable adhesion properties of reactive acrylic copolymers resulting from cross-linking and gas evolution. *The Journal of Adhesion*.

[8] Asbeck A. T., Kim S., Cutkosky M. R., Provancher W. R., Lanzetta M. (2006). Scaling hard vertical surfaces with compliant microspine arrays. *The International Journal of Robotics Research*.

[9] Lee G., Kim H., Seo K., Kim J., Sitti M., Seo T. W. (2016). Series of multilinked caterpillar track-type climbing robots. *Journal of Field Robotics*.

[10] Xu F., Shen J., Hu J. L., Jiang G. P. (2016). A rough concrete wall-climbing robot based on grasping claws: mechanical design, analysis and laboratory experiments. *International Journal of Advanced Robotic Systems*.

[11] Wang Z., Ding X., Rovetta A., Giusti A. (2011). Mobility analysis of the typical gait of a radial symmetrical six-legged robot. *Mechatronics*.

[12] Schmid D., Maeule B. Tracked robot goes up the wall.

[13] Zhang H., Zhang J., Liu R., Zong G. (2005). Realization of a service robot for cleaning spherical surfaces. *International Journal of Advanced Robotic Systems*.

[14] Howard D., Zhang S. J., Sanger D. J. (1996). Kinematic analysis of a walking machine. *Mathematics and Computers in Simulation*.

[15] Shkolnik A., Tedrake R. Inverse kinematics for a point-foot quadruped robot with dynamic redundancy resolution.

[16] García-López M. C., Gorrostieta-Hurtado C., Vargas-Soto E., Ramos-Arreguín J. M., Sotomayor-Olmedo A., Morales J. C. M. (2012). Kinematic analysis for trajectory generation in one leg of a hexapod robot. *Procedia Technology*.

[17] Campa R., Bernal J., Soto I. Kinematic modeling and control of the hexapod parallel robot.

[18] Xin G., Deng H., Zhong G., Wang H. (2015). Hierarchical kinematic modelling and optimal design of a novel hexapod robot with integrated limb mechanism. *International Journal of Advanced Robotic Systems*.

[19] Soyguder S., Alli H. (2012). Kinematic and dynamic analysis of a hexapod walking–running–bounding gaits robot and control actions. *Computers and Electrical Engineering*.

[20] Liu T.-H., Jiang S.-H. (2013). Stability analysis and simulation of bionic hexapod robot. *Computer Simulation*.

[21] Long S., Xin G., Deng H., Zhong G. (2015). An improved force-angle stability margin for radial symmetrical hexapod robot subject to dynamic effects. *International Journal of Advanced Robotic Systems*.

[22] Roy S. S., Pratihar D. K. (2012). Effects of turning gait parameters on energy consumption and stability of a six-legged walking robot. *Robotics and Autonomous Systems*.

[23] Gui B., Wang H., Chen W. Stability analysis for a hexapod robot walking on slopes.

[24] Zhang C., Jiang X., Teng M., Teng J. Research on gait planning and static stability of hexapod walking robot.

[25] Sandoval-Castro X. Y., Castillo-Castaneda E., Lozano-Guzman A. A. Hexapod walking robot CG analytical evaluation.

[26] He B., Xu S., Zhou Y., Wang Z. (2018). Mobility properties analyses of a wall climbing hexapod robot. *Journal of Mechanical Science and Technology*.

[27] Ugurlu B., Kawamura A. (2010). ZMP-based online jumping pattern generation for a one-legged robot. *IEEE Transactions on Industrial Electronics*.

[28] Caron S., Pham Q. C., Nakamura Y. (2017). ZMP support areas for multicontact mobility under frictional constraints. *IEEE Transactions on Robotics*.

[29] Kim D. W., Kim N.-H., Park G.-T. (2012). ZMP based neural network inspired humanoid robot control. *Nonlinear Dynamics*.

[30] Shin H.-K., Kim B. K. (2014). Energy-efficient gait planning and control for biped robots utilizing the allowable ZMP region. *IEEE Transactions on Robotics*.

[31] He B., Xu S., Wang Z. (2018). A novel stiffness model for a wall-climbing hexapod robot based on nonlinear variable stiffness. *Advances in Mechanical Engineering*.

